# Evaluation of enzymatically hydrolyzed poultry byproduct meal effects on fecal microbiota and pressure variables in elderly obese cats

**DOI:** 10.3389/fvets.2025.1530260

**Published:** 2025-03-21

**Authors:** Leonardo A. Príncipe, Pedro H. Marchi, Cinthia G. L. Cesar, Andressa R. Amaral, Kelly K. S. Duarte, Gabriela L. F. Finardi, Jennifer M. Souza, Júlio C. C. Balieiro, Thiago H. A. Vendramini

**Affiliations:** ^1^Pet Nutrology Research Center, Department of Animal Nutrition and Production, School of Veterinary Medicine and Animal Science, University of São Paulo, Pirassununga, Brazil; ^2^Veterinary Nutrology Service, Veterinary Teaching Hospital, School of Veterinary Medicine and Animal Science, University of São Paulo, São Paulo, Brazil

**Keywords:** aldosterone, angiotensin converting enzyme, blood pressure, gut health, microbiome

## Abstract

Arterial hypertension is influenced by the intestinal microbiota and its metabolites, which play a crucial role in host health. Dietary peptides are multifunctional molecules with therapeutic potential for managing hypertension. This study aimed to evaluate the impact of incorporating enzymatically hydrolyzed poultry byproduct meal (EHPM-c) into extruded dry diets on the fecal microbiota and blood pressure parameters of elderly obese cats. Eighteen owners of neutered, clinically healthy male and female cats of various breeds were randomly assigned to two groups: control (30.8%, conventional poultry byproduct meal—CPM-c) and test (17.07%, CPM-c + 12.0% EHPM-c). Clinical values of systolic blood pressure, serum aldosterone concentrations, angiotensin-converting enzyme I activity, and fecal microbiota using 16S rRNA were measured. Data were processed using SAS software (PROC MIXED, PROC GLIMMIX, and PROC CORR; *p* < 0.05). Both groups exhibited high microbial alpha diversity, with no significant differences in beta diversity. Although the inclusion of 12.0% EHPM-c had no measurable effect on blood pressure, both diets promoted beneficial modulation of the fecal microbiota, improving intestinal health. These findings underscore the importance of diet in maintaining gut homeostasis in obese senior cats. While the inclusion of 12.0% EHPM-c did not significantly alter blood pressure parameters, the modulation of the fecal microbiota suggests a potential role in maintaining intestinal health. These results highlight the need for further studies to explore different inclusion levels and longer intervention periods.

## Introduction

Obesity is one of the most prevalent comorbidities diagnosed in the growing cat population. In felines, obesity is defined as an accumulation of body fat exceeding 30% of the ideal weight (1, 2) and it is associated with various diseases, including type II diabetes mellitus (3, 4), hepatic lipidosis (5), lower urinary tract disease (6, 7), dermatological conditions (8), and cardiovascular disorders (9). Furthermore, Chiang et al. (10) reported greater improvements in insulin resistance, diabetes, hyperlipidemia, and dysbiosis in overweight and obese cats. This relationship is well documented in dogs and cats, where obesity-induced dysbiosis contributes to the development of metabolic and inflammatory disorders (11, 12).

Gérard (13) defined dysbiosis as a microbial imbalance linked to the development of disease. Dysbiosis has been extensively studied in humans and is implicated in the pathogenesis of gastrointestinal, metabolic, and cardiovascular conditions, including hypertension (13–18). Thus, the influence of the intestinal microbiota and its metabolites on the etiology, pathophysiology, and mechanisms underlying arterial hypertension has been studied to better understand the condition in humans (19, 20). For example, Li et al. (21) conducted a study using fecal microbiota transplantation from humans into germ-free mice. Fecal samples from normotensive, pre-hypertensive, and hypertensive patients were transplanted into microbiota-free mice. The authors observed differences in bacterial composition among the patient groups and found that the mice developed hypertension after receiving microbiota from hypertensive patients, demonstrating a close relationship between the intestinal microbiota and hypertension. However, the relationship between intestinal dysbiosis and hypertension is not entirely clear.

Arterial hypertension is frequently diagnosed in elderly cats and/or with comorbidities, such as chronic kidney disease, hyperthyroidism, and dilated cardiomyopathy. Regardless of the cause, hypertensive patients are at risk of tissue damage to adjacent organs (22, 23). The treatment aims not only to control blood pressure (BP) values but also to avoid such tissue injuries to the eyes, brain, kidneys, and heart (23). In felines, arterial hypertension is a common condition in elderly animals or those with comorbidities such as chronic kidney disease, hyperthyroidism, or dilated cardiomyopathy (22, 23). Hypertension increases the risk of organ damage, including injuries to the eyes, brain, kidneys, and heart. Current treatment strategies primarily involve the use of amlodipine besylate, a calcium channel blocker (22, 24). However, treatment resistance often develops due to the activation of the renin–angiotensin–aldosterone system (RAAS), necessitating the search for alternative or complementary therapeutic strategies (25).

New non-drug alternatives are needed to controlling hypertension in cats. Therefore, diet peptides emerge as an alternative for controlling BP levels (26). These peptides have an impact on the intestinal microbiota and influence the state of eubiosis (27). In this way, the reduction in arterial hypertension could be linked to the decrease in dysbiosis of the intestinal microbiota (28) as these molecules can modulate and rebalance the intestinal microbiota and, thus, reduce oxidative stress and inflammation in the body and have an antihypertensive effect (29). Furthermore, these molecules perform several functions in the body, as they connect to the receptors of the body and are efficient in inhibiting angiotensin I-converting enzyme (ACE I) activity (30).

Given the need for innovative therapeutic approaches for hypertensive felines, this study aimed to evaluate the effects of incorporating EHPM-c in extruded dry diets for obese senior cats. Specifically, the study assessed changes in fecal microbiota composition and blood pressure variables. It was hypothesized that EHPM-c could alleviate intestinal dysbiosis in obese cats while providing beneficial blood pressure modulation in elderly felines.

## Materials and methods

### Ethical approval

This study was carried out in agreement with the Ethical Principles in Animal Research established by the Ethic Committee on Animal Use of the School of Veterinary Medicine and Animal Science at the University of São Paulo (CEUA/FMVZ). The study was approved under protocol number 8609280422.

### Methodology

The sample size chosen for this study was based on previous clinical nutrition studies, which have utilized a similar number of cats, including studies with obese cats (31–33), cardiovascular research (25, 34–38), and one study with the same thematic (39).

### Study design

The experiment was conducted at the Pet Nutrology Research Center (CEPEN Pet) of the Animal Nutrition and Production Department of the School of Veterinary Medicine and Animal Sciences—University of São Paulo (FMVZ/USP), in the city of Pirassununga, São Paulo, Brazil. Eighteen owners of male (*n* = 12) and female cats (*n* = 6), mixed breed, neutered, with a mean age of 8.46 ± 0.69 years, a mean body score condition (BSC) (40) of 8.71 ± 0.19, and clinically healthy without associated comorbidities were included.

The BSC of the cats was assessed using the 9-point scale developed by Laflamme et al. (40), a validated method routinely employed in veterinary clinical practice. This evaluation involved both visual inspection and palpation of specific anatomical regions, including the ribs, lumbar region, and abdominal area (waist and abdominal recess) with significant fat deposits, as described by Laflamme (40). The presence of subcutaneous fat, waist definition, and abdominal tuck were also carefully assessed to assign a BSC score. BSC evaluations were conducted every 15 days throughout the experimental period to monitor the animals’ condition, ensure the maintenance of BSC values between 8 and 9/9, and determine the need for adjustments in food quantity when necessary.

The animal’s health was previously assessed through a complete physical examination, nutritional anamnesis, complete blood count, and biochemical profile tests [albumin, glucose, total protein, urea, creatinine, alkaline phosphatase, cholesterol, triglycerides, aspartate aminotransferase (AST), and alanine aminotransferase (ALT)] in order to assess the health status of the animals. Other comorbidities, aside from obesity, that could contribute to weight gain were ruled out to check that the cats participating in the project were obese only due to a positive energy balance from excess food.

The experimental design used was completely randomized; that is, the animals were randomly distributed into two experimental groups: the control group (CG; four males and three females) and the test group (TG; six males and three females), which underwent different treatments that occurred simultaneously. The study lasted a total of 77 days, and two periods: the first 30 days were intended for diet standardization, and the 31st day was destined for sample initial collection: the blood pressure of cats was measured using the vascular Doppler method, 5 mL of blood samples was collected from the jugular venipuncture for measurement of serum aldosterone (ALD) concentrations, and testing of ACE I activity and microbiota analysis sterile rectal swab samples were collected with Stuart culture medium. From days 32 to 76, the second period of 45 days began, with the conventionally processed poultry offal meal (CPM-c) and EHPM-c diets. Finally, on day 77, new samples were collected.

### Diets

Two experimental dry diets were extruded: the control diet, with 30.80% CPM-c inclusion and the test diet, with 17.07% CPM-c and 12.00% EHPM-c inclusion (Table 1). Energy intake for each animal was estimated at 130 kcal × body weight 0.4 a day (41).

**Table 1:** Ingredient composition (%) and chemical composition (%) of experimental foods.

Chemical composition (%DM)	Diets	
	Control (CPM-c)	Test (EHPM-c)
Crude protein	33.00	33.00
Ethereal extract	15.00	15.00
Non-nitrogenous extractives	34.03	34.31
Mineral matter	5.83	4.79
Raw fiber	1.40	1.40
Calcium	1.09	0.75
Phosphorus	0.91	0.67
Metabolizable energy (kcal/kg)	4.203	4.203

Ingredients: Conventional poultry offal meal (CPM-c); hydrolyzed poultry offal meal (EHPM-c); corn gluten (60% protein); rice crumb; beet pulp; palatalize powder; poultry offal oil; fish oil; potassium chloride; sodium chloride (common salt); acidifying additive; taurine; mineral and vitamin premix; DL methionine 99%; synthetic antioxidant. Composition of mineral and vitamin premix: vitamin A: 38000 IU/kg, vitamin B1: 38.27 mg/kg, vitamin B12: 4800mcg/kg, vitamin B2: 19.63 mg/kg, vitamin B6: 27.87 mg/kg, vitamin D3: 171000 IU/kg, vitamin E: 535,250 mg/kg, vitamin K: 2.38 mg/kg, pantothenic acid: 49,290 mg/kg, folic acid: 2,480 mg/kg, B.H.T.: 5000 mg/kg, biotin: 1.52 mg/kg, copper: 14.84 mg/kg, choline: 5.157 g/kg, iron: 168.87 mg/kg, iodine: 1.87 mg/kg, manganese: 17.41 mg/kg, niacin: 109.50 mg/kg, selenium: 0.5 mg/kg, zinc: 167.45 mg/kg. Antioxidant composition: BHT: 100 g/kg, citric acid: 35 g/kg, and BHA:10 g/kg.

### Microbiota analysis

Rectal swab samples were collected aseptically using Stuart culture medium. Subsequently, the swabs were stored and frozen at −80°C, following a methodology adapted from Kieler et al. (12). The microbial community sequencing analysis was conducted at the School of Animal Science and Food Engineering of the University of São Paulo (FZEA/USP), Pirassununga, SP, Brazil.

Sequence reads were demultiplexed using BaseSpace® (Illumina). Read quality was assessed with FastQC software, considering a quality score greater than 30. The 16S rRNA gene sequence data were analyzed using DADA2 package, version 1.24.0 (42), in R software, version 4.4.1. Primers were truncated and filtered with the filterAndTrim function according to quality, chimeric sequences were removed, and taxonomy was assigned to amplicon sequence variants (ASVs) based on SILVA database, version 138.1 (43). Microbiota diversity analyses were performed using PhyloSeq package, version 1.38 (44), and MicroEco package, version 1.9.2 (45).

### Blood pressure measurement

Blood pressure measurements were conducted in the morning, during the animals’ routine monitoring sessions between 7:00 AM and 10:00 AM. The procedure followed the guidelines established by the 2018 Consensus Statements of the American College of Veterinary Internal Medicine on systemic hypertension in dogs and cats (23). Measurements were standardized and performed in a quiet, isolated environment. Animals were not sedated and were allowed to acclimate to the room for 5–10 min prior to the assessment.

The BP of cats was assessed using the vascular Doppler method. The animal was positioned in right lateral decubitus, and a cuff appropriate for the patient’s size was positioned on the left anterior limb, specifically in the mid-distal portion of the radius and ulna. Trichotomization of the palmar metacarpal region was performed above the pad where the pulse could be palpated. Gel was applied to the region to facilitate the insertion of the transducer. Utilizing a portable vascular Doppler, pulse signals in the region were identified. Once a stable sound was detected, the cuff was inflated by the sphygmomanometer until the pulse ceased (approximately 200 mmHg) and then slowly deflated. Systolic blood pressure (SBP) was determined at the moment the pulse signal became audible again (23).

The cats were gently restrained in a comfortable position, typically in ventral or lateral recumbency, to minimize the vertical distance between the heart base and the cuff. All BP measurements were performed by the same trained individual, following a consistent protocol, while the animal was calm and motionless. The first measurement was discarded, and five to seven consecutive consistent values were recorded. The final BP value was obtained by averaging these measurements.

### Serum aldosterone concentrations

For initial and final assessments of aldosterone levels, 2 mL of blood was collected via puncture of the jugular or cephalic vein. The collected blood was placed in 5-mL tubes (BD Vacutainer, São Paulo, SP, Brazil), containing clot-activating gel. To ensure optimal coagulation, the samples were allowed to rest for 2 h at room temperature before being centrifuged for 15 min at 1,000 x g (or 3,000 rpm) at 2 ~ 8°C. The resulting supernatant serum was then transferred to labeled plastic tubes and stored at −80°C until analysis. Aldosterone levels were determined using the Aldosterone ELISA Kit provided by the Laboratory Specialized in Scientific Analysis in São Paulo, which has been duly validated for this species. All determinations were performed in the same laboratory.

### Testing angiotensin I-converting enzyme activity

For initial and final assessments of angiotensin I-converting enzyme activity, 2 mL of blood was collected via puncture of the jugular or cephalic vein. The collected blood was placed in 5-mL tubes (BD Vacutainer, São Paulo, SP, Brazil) containing clot-activating gel. Samples were centrifuged for 20 min at 1000 x g (or 3,000 rpm) at 4°C within 30 min after collection. The resulting supernatant serum was transferred to labeled plastic tubes and stored at −80°C until analysis. Serum ACE activity was measured using the fluorimetric method as described by Yang and Neff (46), with some modifications. Briefly, triplicate serum samples (10 μL) were incubated for 30 min at 37°C with 490 μL of ACE and 5 mM of buffered hippuryl-histidyl-leucine solution. Following incubation, 1.2 mL of 0.34 N NaOH was added to stop the reaction. Subsequently, 100 μL of 2% o-phthaldialdehyde in methanol was added and incubated for 10 min. The reaction was halted by adding 200 μL of 3 N HCl. The tubes were then centrifuged at 3000 rpm for 10 min. The supernatant was read using a spectrophotometer (Shimadzu, RF-1501, Kyoto, JP) with an excitation wavelength of 365 nm and an emission wavelength of 495 nm. ACE activity was expressed in units of enzymatic activity per liter of serum (U/L). These analyses were conducted at the Laboratory Specialized in Scientific Analysis in São Paulo.

### Statistical analyses

All findings from this study were assessed by examining individual effects and those linked to the experimental diet. To evaluate quantitative variables, a general linear mixed model was employed, incorporating fixed effects such as treatment (control and test), time (T0 and T45), treatment–time interaction, and random effects of animal and residue. Repeated measurements were conducted within the same experimental units; that is, the same animals were evaluated at two distinct time points. The covariance structures between repeated measurements were determined using the Akaike information criterion (47). Assumptions of the analysis of variance models, including normality of residuals and homogeneity of variances, were simultaneously assessed via analysis of studentized conditional residuals. Statistical analyses were conducted using the PROC MIXED and GLIMMIX procedures within Statistical Analysis System program, version 9.4 (SAS Institute Inc., Cary, NC, USA). Correlations between microbiota and other variables were examined using the PROC CORR procedure, with significance set at *p <* 0.05.

## Results

Sixteen cats completed the clinical trial (GC: 7 cats and TG: 9 cats) (Table 2). Two cats from the control group were excluded due to the owners’ non-compliance with the study protocols.

**Table 2:** Baseline characteristics of control and test groups, including age, body weight, body condition score, muscle mass score, and food consumption.

Variable	Treatment	Time		SE	*p*-value		
		T0	T45		TREAT	TP	TREAT*TP
Age	Control	8.43	8.43	0.69	0.4135	1.00000	1.00000
	Test	7.89	7.89	0.61			
BW	Control	5.80	5.96	0.29	0.3510	0.61480	0.94970
	Test	6.08	6.20	0.26			
BSC	Control	8.71	8.71	0.19	0.1493	1.00000	1.00000
	Test	8.44	8.44	0.17			
MMS	Control	2.71	2.17	0.19	0.7891	1.00000	1.00000
	Test	2.67	2.67	0.17			
FC	Control	61.96	61.96	1.26	0.0756	1.00000	1.00000
	Test	64.15	64.15	1.11			

BW, body weight; BSC, body score condition; CPM-c, conventional poultry byproduct meal; EHPM-c, enzymatically hydrolyzed poultry byproduct meal; FC, food consumption; MMS, muscle mass score; SE = standard errors; TREAT = treatment; TP = time point; TREAT*TP: treatment x time point.

### Fecal microbiota

**Figure 1:**
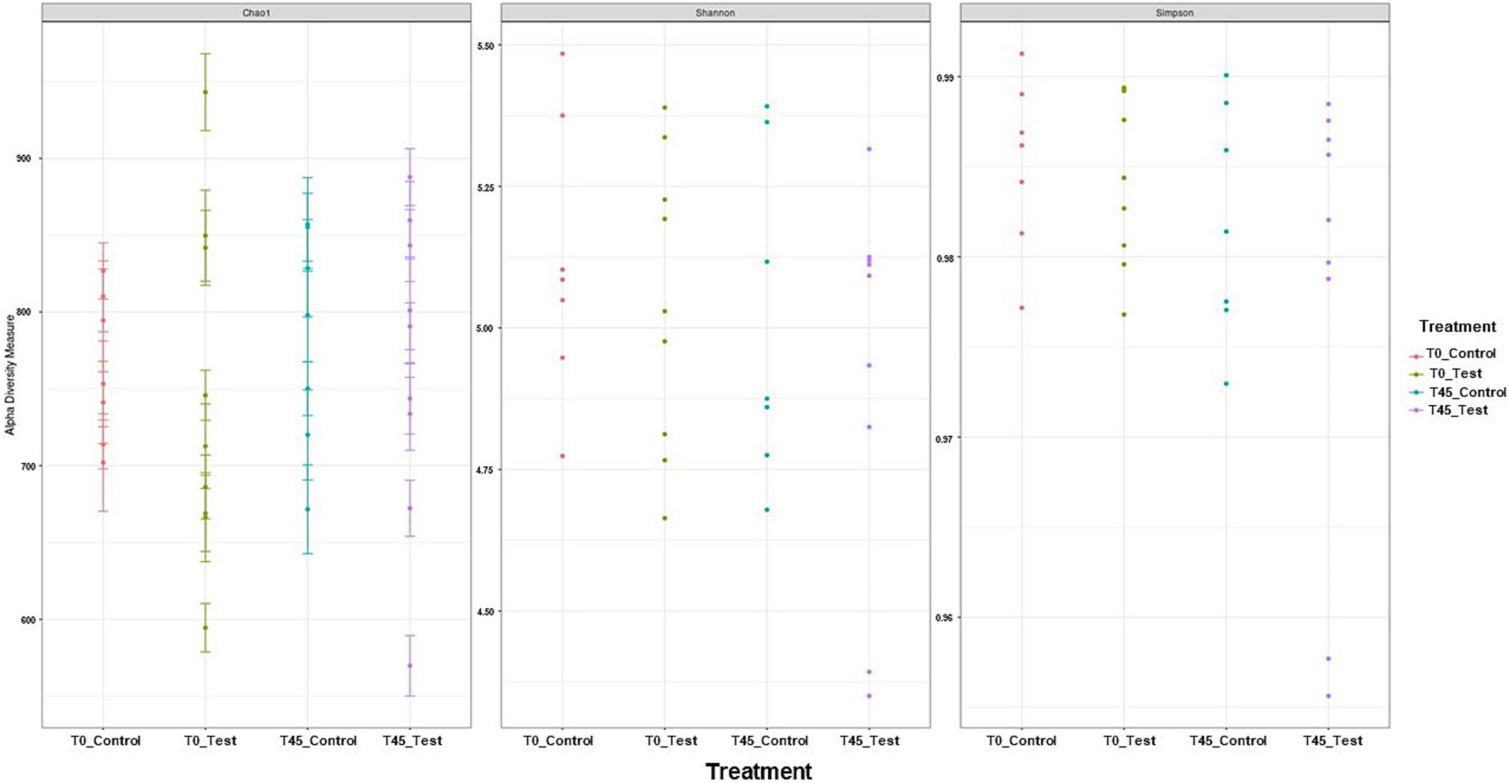
Alpha diversity analyses of the microbial community in experimental groups using Shannon–Wiener, Simpson, and Chao1 indices.

**Figure 2:**
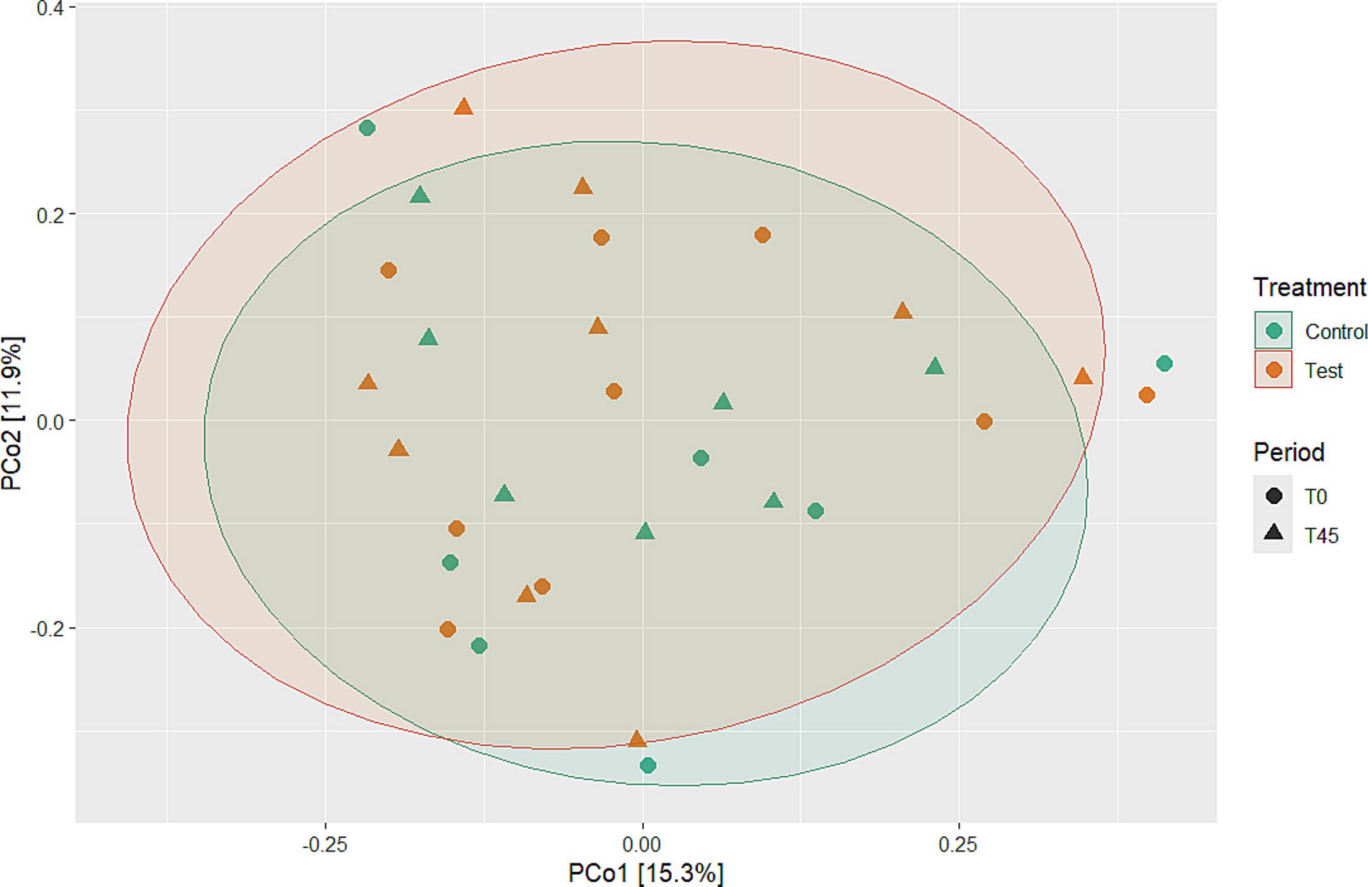
Beta diversity analyses using PCoA to evaluate microbial community differences between experimental groups over time and treatment.

Among the results obtained by sequencing the animals’ fecal microbiota and bioinformatics analysis, were classified 1,414 ASVs to kingdom level; 1,365 for phylum; 1,344 for class; 1,312 for order; 1,237 for family, 977 for genus, and 4 for species. Regarding the alpha diversity indexes evaluated in this study, high microbiota diversity was found through the Shannon (5.02 ± 0.28, *p =* 0.9286) and Simpson (0.98 ± 0.01, *p =* 0.5903) indexes, and adequate abundance using the Chao1 index (764.94 ± 85.95, *p =* 0.9695) for individual samples for both groups (48) (Figure 1; Table 3). Beta diversity was analyzed to assess differences in the composition and structure of the microbial community (Figure 2) and revealed that the treatment did not influence the microbiota.

**Table 3:** Alpha diversity indices (Chao1, Shannon, and Simpson) of microbiota.

Variable	Time	Treatment		SE	*p*-value		
		CPM-c	EHPM-c		TREAT	TP	TREAT*TP
Chao1	T0	763.09	745.45	33.76	0.6801	0.3352	0.9695
	T45	782.92	766.89	29.77			
Shannon	T0	5.12	5,04	0.11	0.4674	0.2359	0.9286
	T45	5.01	4.92	0.10			
Simpson	T0	0.985	0.984	0.003	0.4317	0.1175	0.5903
	T45	0.982	0.978	0.003			

CPM-c, conventional poultry byproduct meal; EHPM-c, enzymatically hydrolyzed poultry byproduct meal; SE = standard errors; TREAT = treatment; TP = time point; TREAT*TP: treatment x time point.

Taxonomic analysis showed that the bacterial communities were dominated by Actinobacteriota, Bacteroidota, Campylobacterota, Cyanobacteria, Deferribacterota, Desulfobacterota, Firmicutes, Fusobacteriota, Proteobacteria, and Spirochaetota at the phylum level. The taxon comparisons between time (T0 x T45) and treatment (CG x TG) effects were performed when the referent taxa were represented and all groups, so that eight phyla, 30 families, and 76 genera had valid values for statistical analysis.

**Figure 3:**
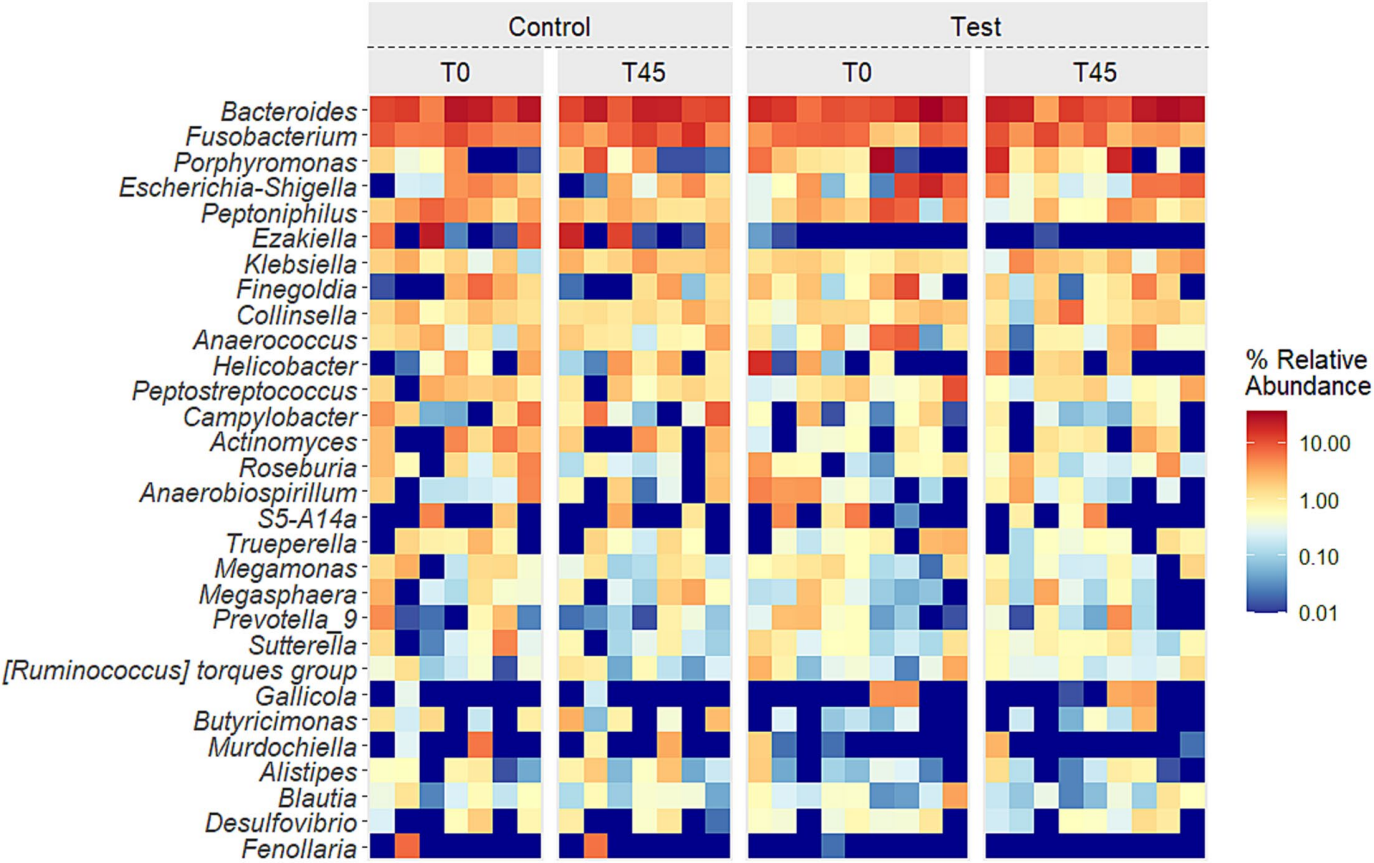
Relative abundance of the 10 principal phyla in the fecal microbiota of experimental groups over time.

No main effect of treatment (between the GC x TG groups) was observed for any of the taxa studied. There was a main effect of time (T0 x T45) for the principal phyla Actinobacteria (*p <* 0.0001), Bacteroidetes (*p =* 0.0272), Campylobacterota (*p <* 0.0001), Firmicutes (*p <* 0.0001), Fusobacteria (*p <* 0.0001), and Proteobacteria (*p =* 0.001) (Figure 3; Table 4).

**Table 4:** Estimates of means and standard errors of the relative abundances of the phyla observed in the study.

Time point								
		T0		T45		*p*-Value		
Phylum		Mean	SE	Mean	SE	TREAT	TP	TREAT*TP
Actinobacteria	Control	5.68^A^	1.15	4.1^B^	0.84	0.2040	<0.0001	<0.0001
	Test	3.15^B^	0.58	3.57^A^	0.65			
Bacteroidetes	Control	25.15^B^	2.09	26.77^A^	2.18	0.0398	<0.0001	0.0272
	Test	32.27^B^	2.14	33.54^A^	2.19			
Campylobacterota	Control	2.44^B^	1.64	2.88^A^	1.93	0.0912	<0.0001	<0.0001
	Test	0.7^A^	0.43	0.36^B^	0.22			
Firmicutes	Control	30.84^A^	3.08	20.62^B^	2.36	0.1967	<0.0001	<0.0001
	Test	24.56^A^	2.36	17.43^B^	1.83			
Fusobacteria	Control	6.71^B^	0.97	8.46^A^	1.20	0.1907	<0.0001	<0.0001
	Test	6.00^A^	0.77	5.58^B^	0.72			
Proteobacteria	Control	24.20^B^	2.12	32.69^A^	2.54	0.7248	<0.0001	0.001
	Test	25.66^B^	1.94	33.41^A^	2.26			

SE = standard errors; TREAT = treatment; TP = time point; TREAT*TP: treatment x time point.

^A,BMeans followed by different letters in the lines differ by 5% in the Tukey–Kramer test adjusted by PROC MIXED.^

Supplementary Table 1 presents the relative abundances of bacterial families in the microbiota, while Supplementary Table 2 contains the relative abundances of the identified microbial genera.

**Figure 4:**
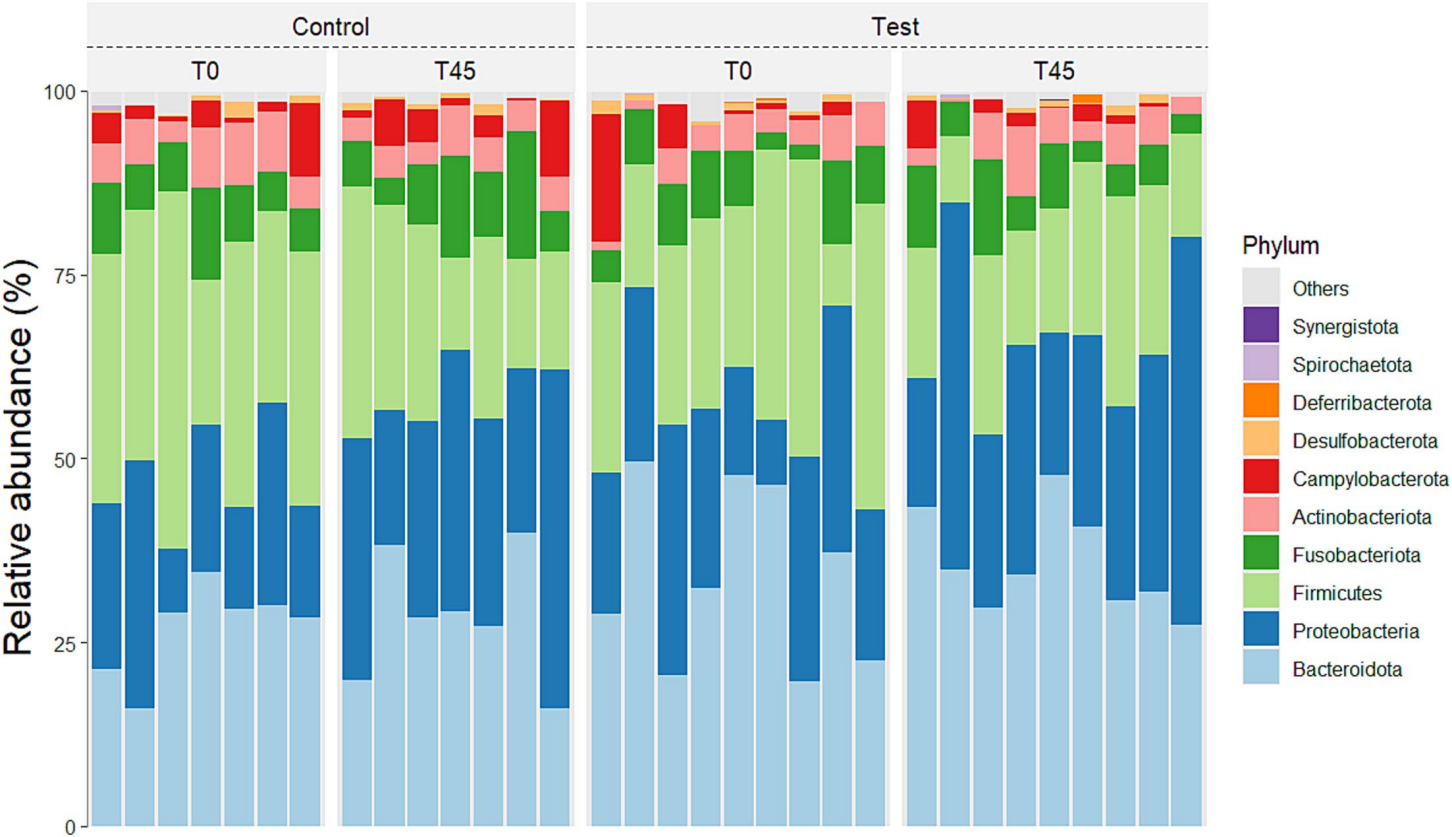
Relative abundance of the 10 principal families in the fecal microbiota of experimental groups over time.

In relation to families, a main treatment effect (T0 x T45) was observed for bacterial groups belonging to the families: *Actinomycetaceae* (*p <* 0.0001), *Aerovoracaceae* (*p =* 0.0014), *Atopobiaceae* (*p <* 0.0001), *Bacteroidaceae* (*p =* 0.0007), *Bifidobacteriaceae* (*p =* 0.0136), *Butyricicoccaceae* (*p <* 0.0001), *Campylobacteraceae* (*p <* 0.0001), *Clostridiaceae* (*p =* 0.0164), *Coriobacteriaceae* (*p =* 0.0002), *Enterobacteriaceae* (*p <* 0.0001), *Enterococcaceae* (*p =* 0.0065), *Erysipelotrichaceae* (*p =* 0.0542), *Family XI* (*p <* 0.0001), *Fusobacteriaceae* (*p <* 0.0001), *Helicobacteraceae* (*p <* 0.0001), *Lachnospiraceae* (*p <* 0.0001), *Marinifilaceae* (*p <* 0.0001), *Mitochondria* (*p =* 0.0021), *Oscillospiraceae* (*p <* 0.0001), *Pasteurellaceae* (*p <* 0.0001), *Peptococcaceae* (*p =* 0.0002), *Porphyromonadaceae* (*p <* 0.0001), *Prevotellaceae* (*p <* 0.0001), *Rikenellaceae* (*p <* 0.0001), *Selenomodaceae* (*p <* 0.0001), *Staphylococcaceae* (*p =* 0.0040), *Succinivibrioceae* (*p <* 0.0001), *Sutterellaceae* (*p <* 0.0001), *Tannerellaceae* (*p =* 0.0421) and *Veillonellaceae* (*p* = 0.1884). There was no main effect of diet consumption (between the CG x TG groups) and no interaction effect (treatment x time; *p* < 0.0001) for the families found (Figure 4; Supplementary Table 1).

**Figure 5:**
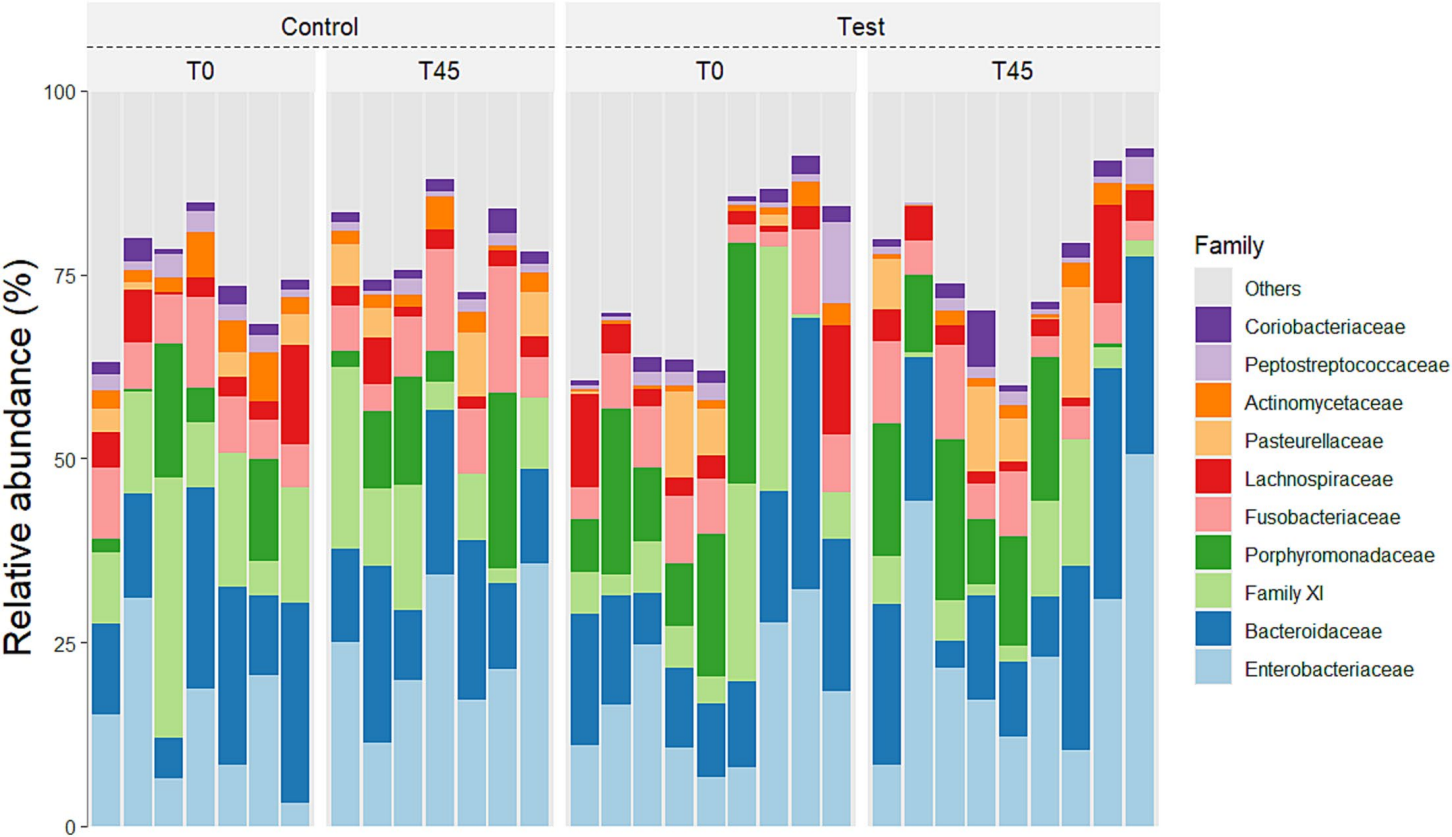
Heatmap of the 30 principal genera in the fecal microbiota of experimental groups over time.

The presents the average relative abundances for the main genera described in literature for cats for which the effect was evaluated in groups (CG x TG) and times (T0 x T45), and only the treatment effect was observed (T0 x T45) (Figure 5; Supplementary Table 2).

The alterations observed in the microbiota highlight its clinical relevance as an indicator of gastrointestinal health and overall wellbeing. The diversity, richness, and abundance of microbial communities, as seen in this study, are consistent with healthy microbiota and can provide insights into the balance and functionality of the feline gastrointestinal system. Variations in specific taxa, such as increases or decreases in key bacterial families, may be associated with diet, health status, or environmental factors, reinforcing the importance of monitoring microbial composition to better understand and support feline health.

### Pressure parameters

Regarding the determination of blood pressure measurement, clinical values indicated that there was no interaction effect for SBP values, serum ALD concentrations, and the ACE I activity test (Table 5).

**Table 5:** Results of blood pressure variables (SBP, ALD, and ACE I).

Pressure parameters		Time point				*p*-value	
		T0	T45	Mean	TREAT	TP	TREAT*TP
SBP	Control	135.00	137.14	5.27	0.413	0.9621	0.7042
	Test	132.78	131.11	4.64			
ALD pg/ml	Control	963.40	1129.83	289.15	0.1469	0.8753	0.6546
	Test	1493.41	1493.31	255.00			
ACE I	Control	26.85	25.80	2.86	0.1906	0.9099	0.7843
	Test	22.50	22.94	2.52			

TREAT = treatment; TP = time point; TRAT*TP: treatment x time point; SBP = systolic blood pressure; ALD = aldosterone; ACE I = angiotensin I-converting enzyme activity; T0: 0 day and T45: 45 days.

## Discussion

Poultry byproduct meal is commonly used in the pet food industry due to its excellent amino acid profile (49), low environmental impact, and reduced cost (50). Enzymatic hydrolysis further enhances its properties by increasing the bioactivity of synthesized molecules (26, 51). Among its benefits, hydrolyzed poultry protein may contain bioactive peptides with the potential to modulate the microbiota (52–54) and inhibit ACE I activity (55–57). While the exact mechanisms by which hydrolyzed proteins influence fecal microbiota composition are not fully elucidated, the diet–microbiota interaction axis is recognized as a critical modulator of host health and disease (58). In particular, dietary peptides derived from protein hydrolysis play a role in maintaining eubiosis (30). Intestinal bacteria preferentially utilize peptides over free amino acids during fermentation (59), which may impact protein fermentation, nutrient absorption, and functional properties within the gut.

In this study, we investigated this potential by assessing the effects of a diet containing EHPM-c in fecal microbiota and serum ACE activity and ALD concentration in elderly obese cats. While the modulation of fecal microbiota showed promising effects, no significant differences were observed in the blood pressure variables analyzed, highlighting the complexity of the mechanisms involved.

Regarding the analysis of fecal microbiota, in the present study, high microbiota diversity and good abundance were found for individual samples from animals in both groups. However, no differences in beta diversity were observed; that is, there were no changes in microbial composition or structure between the experimental groups (60). This result suggests that the study animals had a homogeneous microbiota. The high diversity and good abundance observed are in accordance with what is described in the literature for healthy cats (61). In contrast, a study by Kathrani et al., 2022 (52) found that cats with chronic enteropathy (CE) exhibited a decrease in *α*-diversity, particularly in genus richness, alongside an increase in *β*-diversity when compared to healthy control cats. The feline gastrointestinal tract (GIT) is composed of a complex phylogenetic diversity of microbial organizations (62); it is believed that felines have a more diverse microbiota when compared to dogs (63). In this way, the greater richness, diversity, and abundance of species can be used as an indicator of healthy microbiota (64). All the animals in this study were obese, some previous studies observed that there was no differentiation in terms of microbiota diversity between healthy and obese cats (65, 66). However, the 30-day diet standardization period was effective in stabilizing the microbiota across all animals. There are no studies that have observed differences in the alpha diversity of feline microbiota at different stages of life (67).

According to what is described in the scientific literature, these are predominant bacterial phyla found in the normal microbiota of the feline GIT (61, 63, 68–71).

The Firmicutes/Bacteroidetes (F/B) ratio serves as an essential biomarker for hypertension in humans and spontaneously hypertensive rats (SHR) models. For both groups, the F/B ratio decreased over time. The decrease in the F/B ratio was caused by an increase in the relative abundance of the phyla Bacteroidetes and a reduction in the relative abundance of Firmicutes. Reduced microbial richness and a significant increase in the F/B ratio in animal models of hypertension suggest the presence of intestinal dysbiosis associated with the condition (72). In our study, we can observe that there was no reduction in microbial richness nor an increase in the F/B ratio.

The Firmicutes phylum is the most abundant in the intestinal microbiota of cats (71, 73, 74). Bacteroidetes and Proteobacteria are the second and/or third most abundant phyla. Minamoto et al. (2008) (61) found that healthy cats’ fecal samples had a high relative abundance of Bacteroidetes. The genera Bacteroides was also observed in greater abundance in the fecal microbiota of felines (73, 75). The increase in Bacteroidetes compared to Firmicutes, in our study, is related to the type of sample used for analysis, as they are samples collected via rectal swab. Proteobacteria which have high species diversity in healthy cats’ fecal microbiota (76, 77) increased in cats fed diets with higher protein content (78). Proteobacteria are related to protein metabolism (76), explaining their abundance in carnivorous species like felines.

The TG showed an increased relative abundance of Campylobacterota and Fusobacteria and a decrease in Actinobacteria. While Campylobacterota includes pathogenic species, it is typically present in low abundance in healthy cats (79). Bojanić et al. (80) found increased Campylobacter spp. in healthy dogs and cats, consistent with our findings. Fusobacteria, a key group in carnivorous animals consuming high-protein diets, increased in TG cats, suggesting a dietary effect (81, 82). The decrease in Actinobacteria likely resulted from the increased abundance of Fusobacteria and Proteobacteria, maintaining the usual feline microbiota composition (76).

These findings align with previous literature. Lyu et al. (83) highlighted the variability in the relative abundance of these bacterial phyla. The phylogenetic predominance observed in this study was consistent with that reported in another study involving cats, independent of dietary factors (84, 85). Environmental factors, lifestyle, and analysis methods, as noted by Deng and Swanson (86), may also contribute to these variations. Despite these shifts, the TG cats maintained a healthy and balanced microbiota. The inclusion of EHPM-c (12.00%) did not significantly alter microbiota composition compared to the CG, although an interaction effect was observed for Actinobacteria, Campylobacterota, Cyanobacteria, Firmicutes, and Fusobacteria.

The *Bacteroidaceae* family, along with the *Bacteroides* genus, showed an increased relative abundance in TG and a decrease in CG. Similar findings were reported by Minamoto et al. (61), who identified *Bacteroides* as a dominant genus in the feline GIT. Suchodolski et al. (87) and Giordano et al. (88) noted a decrease in *Bacteroidaceae* and *Bacteroides*, respectively, in cats with enteropathy. However, the increase observed in TG suggests a beneficial impact of the EHPM-c diet. In contrast, no changes were found for *Bifidobacteriaceae* or *Bifidobacterium* in TG, although the CG observed a decrease. Decreases in *Bifidobacterium* are commonly observed in cats with chronic enteropathy (88, 89).

The TG diet reduced the abundance of *Campylobacteraceae* and *Campylobacter*, whereas the CG showed an increase. Campylobacter is a common enteropathogen, and increased abundance can indicate dysbiosis (87, 90). No changes were observed in *Coriobacteriaceae* or *Collinsella*, but both were more abundant in TG. Jia et al. (67) found higher *Coriobacteriaceae* levels in elderly cats. *Enterobacteriaceae* increased in both groups, but *Escherichia-Shigella* and *Clostridium sensu stricto 1* decreased in TG, consistent with findings in obese cats and those with inflammatory bowel disease (91, 92).

Both groups observed a reduction in *Erysipelotrichaceae* and *Lachnospiraceae* at T45, with a corresponding increase in *Roseburia* in TG and a decrease in CG. Such reductions are often seen in obese animals (93). Increased concentrations of *Lachnospiraceae* have been associated with cases of chronic hemorrhagic diarrhea (87). *Roseburia* is typically elevated in obese dogs (94), but no similar increase has been reported in cats.

The EHPM-c diet also decreased *Fusobacteriaceae*, *Helicobacteraceae*, and *Pasteurellaceae* in TG, whereas their abundance increased in CG. *Fusobacterium* and *Helicobacter*, part of the normal feline microbiota (61, 80), decreased in TG. *Fusobacterium*, when increased, have been associated with dysbiosis and intestinal lymphoma in cats (88, 95, 96), while increased *Pasteurellaceae* correlates with dysbiosis (61, 68, 97). The reduction in *Helicobacter* has been reported in cases of diarrhea (98).

Finally, the TG showed increased relative abundance of *Staphylococcaceae*, while no changes were seen in CG. Although *Staphylococcus* is present in healthy cats (61, 68), it can be pathogenic in humans (86, 87). Interestingly, Garcia-Mazcorro et al. (74) observed increased *Staphylococcaceae* after prebiotic supplementation in dogs but not in cats. *Veillonellaceae* increased in TG and decreased in CG, as observed with other genera in the family. *Veillonellaceae* is significant in the fecal microbiome of both dogs (63) and cats (30), and its increase has been linked to prebiotic use (74).

Regarding the analysis of pressure variables, there was no difference between the groups to SBP values, the mean SBP was 133.80 ± 13.40 mmHg, indicating the animals’ health and normal blood pressure. Serum ALD concentrations were analyzed to compare mean levels between experimental groups and explore the potential antihypertensive effects of EHPM-c. ALD is a hormone responsible for maintaining the sodium and potassium balance of the body and therefore acts on the control of extracellular volume (99). Vasoconstriction by angiotensin II (ANG II) stimulates the release of ALD, increasing blood volume and SBP.

ACE I catalyzes this process (100, 101). It was hypothesized that serum ALD concentrations would decrease in cats fed EHPM-c due to inhibition of ACE I action. However, serum ALD concentrations did not differ between treatments and times.

In the pharmacological treatment for feline arterial hypertension, it was observed that even when using inhibitor ACE I medication, serum ALD concentrations in chronic kidney patients with hypertensive cats did not decrease (34). Previous studies demonstrated that there was no difference in serum ALD concentration between normotensive and hypertensive cats (102, 103). Jensen et al. (104) observed that there was no change in ALD concentrations in felines after treatment with amlodipine. The lack of significance in serum ALD concentrations between the groups in this study may reflect the action of alternative pathways and mechanisms for maintaining ALD levels in the blood.

Neurohormonal mechanisms may potentially prevent the decline of hormones such as ALD, thereby maintaining intravascular volume. These effects may be attributed to alternative pathways for generating ANG II (34). There is the possibility of the presence of an alternative RAAS pathway, in which the enzyme angiotensin II-converting enzyme (ACE II), homologous to ACE I, catalyzes angiotensin I (ANG I) and ANG II into peptides with opposite effects (e.g., ANG1-7) responsible for causing vasodilation, diuresis, natriuresis, and attenuate vascular inflammation (105, 106). In this way, the alternative RAAS pathways act in a counter-regulatory manner and reduce the negative effects of ANG II and ALD. For this reason, serum concentrations of biomarkers and molecules do not determine the final result of RAAS. This effect is at the mercy of their interaction with their respective target receptors.

In order to evaluate the inhibitory potential of EHPM-c on ACE I, the ACE I activity test was carried out in the experimental groups and the mean levels were verified. ACE is responsible for the conversion of ANG I and ANG II and, therefore, controls the vasoconstrictive and sodium-retaining properties of ANG II, which are related to BP (107). Therefore, inhibitor ACE I agents are studied to control SBP in cats (107–109). Through the antihypertensive effects of dietary peptides, a decrease in ACE I activity in cats fed EHPM-c was hypothesized. However, serum ACE I activity did not differ between treatments and times.

According to literature, cats have higher serum ACE I activity (12.7 ± 1.0 mU/mL) than dogs (5.9 ± 0.6 mU/mL) (110). In this way, it was hypothesized that activation of alternative RAAS pathways could contribute that there are no changes in the serum concentrations of ACE I and other components (105). Importantly, in our study, all cats were clinically healthy and normotensive. It is believed that through the feedback mechanism, ANG II can regulate the levels of ACE I activity as well as influence the expression and activity of the alternative ACE II pathway (111). Therefore, agent inhibitor ACE I may not be effective in suppressing the renin–angiotensin system (112). Furthermore, ANG II synthesized by alternative routes, chymostatin-sensitive angiotensin II-generating enzyme (CAGE) and/or ACE II, is inert to the action of ACE I inhibitors (113, 114). However, serum ANG II concentrations were not evaluated in the present study. Furthermore, as with serum ALD concentrations, the organism uses compensatory mechanisms and alternative pathways for which serum RAAS components do not change in healthy individuals. Studies are needed to evaluate the activity of target receptors and biomarkers in the body.

The prevalence of obesity among companion animals is an increasingly concerning issue. This problem manifests differently across species. For example, obesity in cage birds, including canaries, is aggravated by the use of commercial egg-based feeds and modern extruded bird diets, along with the absence of standardized energy requirements (115). For this reason, the inclusion of novel ingredients, such as byproducts, in pet food formulations has been explored as a strategy to enhance digestibility and nutritional value (116). This approach holds potential for further exploration, particularly as adjuncts in the management of various comorbidities in dogs and cats. Thus, obesity was selected in this study as a model of dysbiosis due to its high prevalence in cats and other species, making it a relevant condition for investigating the impact of hydrolyzed poultry byproduct meals on the microbiota of obese animals.

The data presented in this study should be interpreted with an understanding of its limitations. The findings may not be applicable to hypertensive cats in general, as the animals used in this study were clinically healthy, despite obesity. The comparison was made exclusively between healthy obese cats, as cats are often used as experimental models for similar or identical diseases found in humans. This approach provides new insights into the etiology, diagnosis, and treatment of both species. The choice of this model is justified by the fact that obesity is a known risk factor for hypertension in humans. As such, this study design limits the interpretation of the findings as to whether no changes noted between groups were due to no hypertensive cats. The conditions for the cats’ participation in the trial required them to be fed only the trial diets, with no additional treats or snacks, which could have influenced their overall nutrient intake. As privately owned cats were included in the study, the variability within the sample population presented a potential risk for confounding effects. However, because there were no significant differences in independent variables such as sex, age, body weight, BSC, season, and breed, along with health and wellness indicators at the start of the study, randomization was deemed successful, minimizing any risk of bias between the groups.

Another limitation of using privately owned animals was the trial duration. The 45-day period for the experimental diets was chosen based on data extrapolated from other species or previous feline studies. However, a longer trial might have revealed additional differences. That said, extending the study duration could introduce challenges related to protocol compliance and retention of client-owned cats. The small sample size, short study duration, and the use of client-owned cats represent significant limitations that warrant further consideration. A larger sample size would enhance the statistical power of the study, reducing the risk of type II errors and improving the generalizability of the findings. The 45-day study duration, while adequate to observe initial effects, might not have been sufficient to capture longer term physiological changes or stabilize microbiota responses. Extending the trial could provide a more comprehensive understanding of the effects of intervention but would also increase challenges related to protocol compliance. Additionally, the inclusion of client-owned cats introduces variability due to differences in home environments, stress levels, and adherence to dietary protocols, which could act as confounding factors despite efforts to standardize conditions and randomize groups.

The inclusion of EHPM-c in the diet of cats impacted the microbiota composition but did not affect blood pressure variables. This finding is understandable given that blood pressure is highly sensitive, and the body has several metabolic pathways to maintain blood pressure and homeostasis. Furthermore, all animals in the study were normotensive, meaning they did not present elevated blood pressure, which may have influenced the lack of changes in the blood pressure variables. It is also important to note that this study focused on obese senior cats, and thus, the results may not be directly applicable to non-obese or younger cats, as the physiological and metabolic differences could affect the response to the inclusion of EHPM-c. This study is pioneering and provides a new perspective on the use of hydrolyzed chicken offal meal in extruded diets for obese senior cats, opening avenues for future research on its effects on various health conditions.

More research is needed to further elucidate the health impact (pressure parameters and fecal microbiota) of enzymatically hydrolyzed poultry byproduct meal in elderly obese cats. Based on these findings, future studies should investigate the application of EHPM-c in persistently hypertensive cats to assess whether this ingredient may have significant effects on both microbiota and blood pressure variables in the context of hypertension. Additionally, the impact of microbiota modulation on the metabolism of these animals could be further understood through the analysis of microbiota metabolites, expanding the understanding of the interactions between intestinal microbiota and the overall health status of felines.

## Conclusion

This study concludes that the inclusion of 12.00% EHPM-c in extruded diets did not influence the blood pressure variables of elderly obese cats. However, both diets proved effective in promoting beneficial modulation of the fecal microbiota, highlighting the potential role of diet in maintaining gut health in this population.

## Data Availability

The data presented in the study are deposited in the NCBI Repository, BioProject Accession Number: PRJNA1226564, https://www.ncbi.nlm.nih.gov/sra/PRJNA1226564.
