# Supplementary material for: Evaluation of enzymatically hydrolyzed poultry byproduct meal effects on fecal microbiota and pressure variables in elderly obese cats
Source: Front Vet Sci. 2025 Mar 21;12:1530260. doi: 10.3389/fvets.2025.1530260 (PMC11969457; doi:10.3389/fvets.2025.1530260)
Supplement: Supplementary file 1 [file Table_1.docx]

| **Supplementary table 1.** Estimates of means and standard errors of the relative abundances of the families observed in the study | | | | | | | | | |
| --- | --- | --- | --- | --- | --- | --- | --- | --- | --- |
|  | | **Time Point** | | | |  | | |  |
|  | | **T0** | | **T45** | | **p-Value** | | |  |
| **Families** |  | **Mean** | **SE** | **Mean** | **SE** | **TREAT** | **TP** | **TREAT*TP** |  |
| *Actinomycetaceae* | Control | 3.274^A^ | 0.7724 | 1.993^B^ | 0.47680 | 7.71 | 96.63 | 341.95 | **<0.0001** |
|  | Test | 0.9739^B^ | 0.2083 | 1.136^A^ | 0.24230 |  |  |  |  |
| *Aerovoracaceae* | Control | 0.47^A^ | 0.2905 | 0.3185^B^ | 0.19572 | 0.24 | 400.51 | 16.25 | **0.0014** |
|  | Test | 0.3464^A^ | 0.1904 | 0.1927^B^ | 0.10610 |  |  |  |  |
| *Atopobiaceae* | Control | 0.0749^A^ | 0.5420 | 0.0079±^B^ | 0.00590 | 0.21 | 104.38 | 52.71 | **<0.0001** |
|  | Test | 0.0456^A^ | 0.0289 | 0.0312^B^ | 0.01970 |  |  |  |  |
| *Bacteroidaceae* | Control | 15.36^A^ | 2.8060 | 14.79^B^ | 2.72100 | 0.00 | 18.55 | 117.79 | **0.0007** |
|  | Test | 14.19^B^ | 2.3180 | 15.48^A^ | 2.49000 |  |  |  |  |
| *Bifidobacteriaceae* | Control | 0.0446^A^ | 0.0197 | 0.0199^B^ | 0.08800 | 1.11 | 10.70 | 12.70 | **0.0136** |
|  | Test | 0.0581^A^ | 0.0289 | 0.0603^A^ | 0.29700 |  |  |  |  |
| *Butyricicoccaceae* | Control | 0.2242^A^ | 0.0761 | 0.0891^B^ | 0.30700 | 0.10 | 51.90 | 74.71 | **<0.0001** |
|  | Test | 0.1179^A^ | 0.0358 | 0.1282^A^ | 0.03880 |  |  |  |  |
| *Campylobacteraceae* | Control | 0.9311^B^ | 0.6923 | 1.099^A^ | 0.81580 | 3.89 | 58.03 | 184.98 | **<0.0001** |
|  | Test | 0.2007^A^ | 0.1249 | 0.1108^B^ | 0.69100 |  |  |  |  |
| *Clostridiaceae* | Control | 0.025^A^ | 0.0161 | 0.0364^A^ | 0.02340 | 0.40 | 23.83 | 40.6 | **0.0164** |
|  | Test | 0.0667^A^ | 0.0464 | 0.0041^B^ | 0.00320 |  |  |  |  |
| *Coriobacteriaceae* | Control | 1.555^A^ | 0.3638 | 1.499^A^ | 0.35080 | 0.22 | 25.34 | 48.36 | **0.0002** |
|  | Test | 1.176^B^ | 0.2439 | 1.478^A^ | 0.30530 |  |  |  |  |
| *Enterobacteriaceae* | Control | 14.21^B^ | 2.2320 | 21.97^A^ | 3.13800 | 0.04 | 5608.76 | 62.27 | **<0.0001** |
|  | Test | 15.48^B^ | 2.1120 | 21.96^A^ | 2.76600 |  |  |  |  |
| *Enterococcaceae* | Control | 0.0186^A^ | 0.0175 | 0.0223^A^ | 0.02090 | 0.00 | 26.98 | 42.11 | **0.0065** |
|  | Test | 0.0459^A^ | 0.0334 | 0.009^B^ | 0.00660 |  |  |  |  |
| *Erysipelotrichaceae* | Control | 0.166^A^ | 0.0693 | 0.0404^B^ | 0.01710 | 0,00 | 199.48 | 51,54 | 0.0542 |
|  | Test | 0.1018^A^ | 0.0377 | 0.0642^B^ | 0.02390 |  |  |  |  |
| *Family XI* | Control | 12.28^A^ | 3.6350 | 9.002^B^ | 2.76400 | 3.7 | 3471.43 | 361.24 | **<0.0001** |
|  | Test | 6.499^A^ | 1.8090 | 3.409^B^ | 0.98030 |  |  |  |  |
| *Fusobacteriaceae* | Control | 6.709^B^ | 0.9702 | 8.463^A^ | 1.20000 | 1.89 | 82.63 | 294.45 | **<0.0001** |
|  | Test | 5.998^A^ | 0.7711 | 5.577^B^ | 0.72000 |  |  |  |  |
| *Helicobacteraceae* | Control | 0.3015^B^ | 0.2966 | 0.3571^A^ | 0.35110 | 0.00 | 130.32 | 367.42 | **<0.0001** |
|  | Test | 0.4786^A^ | 0.5104 | 0.2455^B^ | 0.26250 |  |  |  |  |
| *Lachnospiraceae* | Control | 3.782^A^ | 1.0000 | 2.164^B^ | 0.58210 | 0.20 | 860.43 | 213.37 | **<0.0001** |
|  | Test | 3.682^A^ | 0.8595 | 3.056^B^ | 0.71810 |  |  |  |  |
| *Marinifilaceae* | Control | 0.381^B^ | 0.2784 | 0.5705^A^ | 0.41610 | 1.63 | 575.2 | 147.38 | **<0.0001** |
|  | Test | 0.0698^B^ | 0.0483 | 0.2402^A^ | 0.16560 |  |  |  |  |
| *Mitochondria* | Control | 4.612^A^ | 0.4425 | 4.276^B^ | 0.41160 | 3.57 | 14.14 | 7.05 | **0.0021** |
|  | Test | 3.507^A^ | 0.3010 | 3.461^A^ | 0.29680 |  |  |  |  |
| *Oscillospiraceae* | Control | 0.803^B^ | 0.0941 | 0.5296^A^ | 0.17660 | 0.28 | 94.01 | 98.08 | **<0.0001** |
|  | Test | 0.4887^A^ | 0.1437 | 0.4855^A^ | 0.14260 |  |  |  |  |
| *Pasteurellaceae* | Control | 0.1762^B^ | 0.2178 | 0.3776^A^ | 0.46570 | 0.00 | 2293.42 | 6.13 | **<0.0001** |
|  | Test | 0.1593^B^ | 0.1842 | 0.3708^A^ | 0.47800 |  |  |  |  |
| *Peptococcaceae* | Control | 0.0794^A^ | 0.3360 | 0.0641^A^ | 0.03200 | 0.44 | 30.24 | 64.6 | **0.0002** |
|  | Test | 0.0266^B^ | 0.0107 | 0.0832^A^ | 0.32700 |  |  |  |  |
| *Porphyromodaceae* | Control | 1.015^B^ | 1.1120 | 1.486^A^ | 1.62100 | 0.17 | 189.25 | 738.46 | **<0.0001** |
|  | Test | 2.366^A^ | 2.2550 | 2.091^B^ | 1.99800 |  |  |  |  |
| *Prevotellaceae* | Control | 0.5452^A^ | 0.3854 | 0.1518^B^ | 0.10780 | 0.00 | 316.57 | 788.15 | **<0.0001** |
|  | Test | 0.2501^B^ | 0.1567 | 0.3331^A^ | 0.20850 |  |  |  |  |
| *Rikenellaceae* | Control | 0.1942^B^ | 0.1233 | 0.3913^A^ | 0.24760 | 1.48 | 136.25 | 56.73 | **<0.0001** |
|  | Test | 0.0908^A^ | 0.5180 | 0.1056^A^ | 0.06020 |  |  |  |  |
| *Selenomodaceae* | Control | 0.665^A^ | 0.3000 | 0.2991^B^ | 0.13560 | 0.00 | 334.96 | 78.51 | **<0.0001** |
|  | Test | 0.5113^A^ | 0.2041 | 0.3873^B^ | 0.15490 |  |  |  |  |
| *Staphylococcaceae* | Control | 0.0165^A^ | 0.0118 | 0.0123^A^ | 0.00890 | 0.76 | 25.35 | 51.13 | **0.0040** |
|  | Test | 0.0027^B^ | 0.0017 | 0.0146^A^ | 0.00880 |  |  |  |  |
| *Succinivibrioceae* | Control | 0.324^A^ | 0.2349 | 0.2418^B^ | 0.17550 | 0.37 | 377.11 | 62.79 | **<0.0001** |
|  | Test | 0.7226^A^ | 0.4865 | 0.3609^B^ | 0.24390 |  |  |  |  |
| *Sutterellaceae* | Control | 0.7232^A^ | 0.2269 | 0.2593^B^ | 0.08210 | 0.19 | 344.87 | 235.64 | **<0.0001** |
|  | Test | 0.5458^A^ | 0.1514 | 0.4952^B^ | 0.13740 |  |  |  |  |
| *Tannerellaceae* | Control | 0.2541^B^ | 0.0607 | 0.3338^A^ | 0.07920 | 0.71 | 5.00 | 21.83 | **0.0421** |
|  | Test | 0.3984^A^ | 0.0732 | 0.3618^A^ | 0.07550 |  |  |  |  |
| *Veillonellaceae* | Control | 0.9786^A^ | 0.4813 | 0.7724^B^ | 0.38070 | 1.20 | 1.91 | 136.03 | 0.1884 |
|  | Test | 0.3632^B^ | 0.1587 | 0.4911^B^ | 0.21420 |  |  |  |  |
| Legend: SE= standard errors; TREAT = treatment; TP = time point; TREAT*TP: treatment x time point.  ^A-B^Means followed by different letters in the lines differ by 5% in the Tukey-Kramer test adjusted by PROC MIXED | | | | | | | | | |
