# Supplementary material for: Evaluation of enzymatically hydrolyzed poultry byproduct meal effects on fecal microbiota and pressure variables in elderly obese cats
Source: Front Vet Sci. 2025 Mar 21;12:1530260. doi: 10.3389/fvets.2025.1530260 (PMC11969457; doi:10.3389/fvets.2025.1530260)
Supplement: Supplementary file 2 [file Table_2.docx]

| **Supplementary table 2.** Estimates of means and standard errors of the relative abundances of the main genera observed in the study | | | | | | | | | |
| --- | --- | --- | --- | --- | --- | --- | --- | --- | --- |
|  | | **Time Point** | | | |  | | |  |
|  | | **T0** | | **T45** | | **p-Value** | | |  |
| **Genera** |  | **Mean** | **SE** | **Mean** | **SE** | **TREAT** | **TP** | **TREAT*TP** |  |
| *Bacteroides* | Control | 15.360^A^ | 2.806 | 14.790^B^ | 2.721 | 0.9461 | 0.0007 | **<0.0001** |  |
|  | Test | 14.190^B^ | 2.318 | 1.136^A^ | 2.490 |  |  |  |  |
| *Bifidobacterium* | Control | 0.045^A^ | 0.020 | 0.020^B^ | 0.009 | 0.3269 | 0.0136 | **0.0090** |  |
|  | Test | 0.058^A^ | 0.029 | 0.060^A^ | 0.030 |  |  |  |  |
| *Blautia* | Control | 0.264^A^ | 0.120 | 0.213^B^ | 0.071 | 0.9024 | <0.0001 | 0.3221 |  |
|  | Test | 0.400^A^ | 0.116 | 0.216^B^ | 0.063 |  |  |  |  |
| *Campylobacter* | Control | 0.931^B^ | 0.692 | 1.099^A^ | 0.816 | 0.0743 | <0.0001 | **<0.0001** |  |
|  | Test | 0.201^A^ | 0.125 | 0.111^B^ | 0.069 |  |  |  |  |
| *Catenibacterium* | Control | 0.091^A^ | 0.054 | 0.077^A^ | 0.046 | 0.5813 | 0.5969 | 0.0750 |  |
|  | Test | 0.047^A^ | 0.022 | 0.063^A^ | 0.030 |  |  |  |  |
| *Clostridium sensu stricto 1* | Control | 0.030^A^ | 0.023 | 0.044^A^ | 0.034 | 0.4679 | 0.019 | 0.0085 |  |
|  | Test | 0.059^A^ | 0.041 | 0.004^B^ | 0.003 |  |  |  |  |
| *Collinsella* | Control | 1.540^A^ | 0.359 | 1.478^A^ | 0.345 | 0.6456 | 0.0003 | **<0.0001** |  |
|  | Test | 1.166^B^ | 0.241 | 1.56^A^ | 0.300 |  |  |  |  |
| *Escherichia-Shigella* | Control | 0.797^A^ | 0.583 | 0.639^B^ | 0.468 | 0.4867 | <0.0001 | **<0.0001** |  |
|  | Test | 1.853^A^ | 1.171 | 1.098^B^ | 0.699 |  |  |  |  |
| *Fusobacterium* | Control | 6.359^B^ | 0.920 | 8.014^A^ | 1.138 | 0.1400 | <0.0001 | **<0.0001** |  |
|  | Test | 5.434^A^ | 0.701 | 5.31^B^ | 0.663 |  |  |  |  |
| *Helicobacter* | Control | 0.302^B^ | 0.297 | 0.357^A^ | 0.351 | 0.9765 | <0.0001 | **<0.0001** |  |
|  | Test | 0.479^A^ | 0.510 | 0.246^B^ | 0.263 |  |  |  |  |
| *Roseburia* | Control | 1.088^A^ | 0.449 | 0.402^B^ | 0.167 | 0.7674 | <0.0001 | **<0.0001** |  |
|  | Test | 0.500^B^ | 0.183 | 0.627^A^ | 0.229 |  |  |  |  |
| *Staphylococcus* | Control | 0.006^A^ | 0.005^A^ | 0.005^A^ | 0.004 | 0.7715 | 0.2342 | 0.1762 |  |
|  | Test | 0.003^A^ | 0.002 | 0.019^A^ | 0.010 |  |  |  |  |
| Legend: SE= standard errors; TREAT = treatment; TP = time point; TREAT*TP: treatment x time point.  ^A-B^Means followed by different letters in the lines differ by 5% in the Tukey-Kramer test adjusted by PROC MIXED | | | | | | | | | |
